# Estimated Impact of Low Isolate Numbers on the Reliability of Cumulative Antibiogram Data

**DOI:** 10.1128/spectrum.03939-22

**Published:** 2023-01-10

**Authors:** Christian Tran, John Hargy, Bryan Hess, Matthew A. Pettengill

**Affiliations:** a Sidney Kimmel Medical College, Thomas Jefferson University, Philadelphia, Pennsylvania, USA; b Department of Pathology, Anatomy, and Cell Biology, Thomas Jefferson University Hospital, Philadelphia, Pennsylvania, USA; c Southern Adventist University, Collegedale, Tennessee, USA; d Division of Infectious Diseases, Thomas Jefferson University Hospital, Philadelphia, Pennsylvania, USA; Johns Hopkins Hospital

**Keywords:** antibiogram, cumulative, stewardship, susceptibility testing

## Abstract

Antibiograms are cumulative reports of antimicrobial susceptibility results that are used to guide the selection of empirical antibiotic therapy. Although Clinical and Laboratory Standards Institute (CLSI) guidelines recommend including only organisms that have at least 30 isolates in an antibiogram, previous studies demonstrated that adherence to this recommendation is highly variable. This paper aims to model the impact of small sample sizes on expected levels of error in cumulative antibiograms by comparing percent susceptibility results for random samples to those of the larger, entire data set. The results demonstrate relatively high error rates when utilizing low numbers of isolates in cumulative antibiograms, and provide a discussion point for considering the appropriate number of isolates that could be utilized, and the impact of increasing isolate numbers by including multiple years of data.

**IMPORTANCE** Antibiograms are reports of local antimicrobial susceptibility patterns for common bacteria and yeast that are used to make empirical decisions for patient therapy and also to inform institution therapy guidelines. This study evaluates the impact of low isolate counts on the reliability of antibiograms, and suggests that more institutions should utilize multiple years of data to overcome this issue.

## INTRODUCTION

An antibiogram is a cumulative report of antimicrobial susceptibility results for common microorganisms produced for a specific population of patients (hospital or other health care facility, region etc.) for a specific period of time (annual or more frequently). When an organism-causing infection has been identified, or there is strong clinical suspicion for a particular organism-causing infection, care providers may consult an antibiogram at their institution to guide selection of empirical therapy. Antibiograms are also useful to inform institution-specific recommendations for empirical antimicrobial therapy. Laboratories that perform antimicrobial susceptibility testing (AST) should generate antibiograms for the institutions that they serve in accordance with Clinical and Laboratory Standards Institute (CLSI) or other official recommendations (1), although several studies have demonstrated that adherence to recommendations is highly variable (2, 3). Current CLSI M39 (5th edition) guidelines recommend that in order “to obtain a reasonable statistical estimate of cumulative [percent susceptibility] %S rates, it is desirable to include only organisms with 30 or more isolates tested during the analysis period” (4). At least one published evaluation of this criteria among 30 hospital antibiograms showed that adherence to this recommendation is poor; Moehring et al. report that only 25% of labs in their study followed the recommendation to exclude organisms with less than 30 isolates (5). We also evaluated 27 recent hospital antibiograms, either publicly available or provided on request, from 19 different medical institutions. Twenty-two of the 27 (81%) included data points for organisms with fewer than 30 isolates, 19 (70%) included data points for organisms with fewer than 20 isolates, and eight (30%) included data points with fewer than 10 isolates. The lowest amount of isolates used for any data point in this set was two. In total, 180 out of 902 (20%) data points from these 27 antibiograms were below 30 isolates (Pettengill, unpublished data).

The expected level of error with a low number of isolates has not been explored in detail in published literature, although it is addressed in the appendices of the CLSI M39 document (pay access) in the form of confidence intervals at various %S and isolate counts. In this study, we used real clinical data from a tertiary hospital for organisms with large AST data sets to simulate the impact of small sample sizes on mean error in cumulative antibiograms to see how this correlates with values predicted by binomial distributions given set %S and sample sizes. In the modeling approach, different sample sizes and randomly selected results from the overall data set were used to predict the mean and maximum errors that may be observed with comparison to true susceptibility rates for the full data set, which correlated well with binomial predictions. Additionally, it demonstrates the potential of using aggregate data from 2 years to calculate a combined susceptibility rate in lieu of single year data, particularly for organisms with low isolate counts.

## RESULTS

### Mean and maximum errors of random samples.

**FIG 1:**
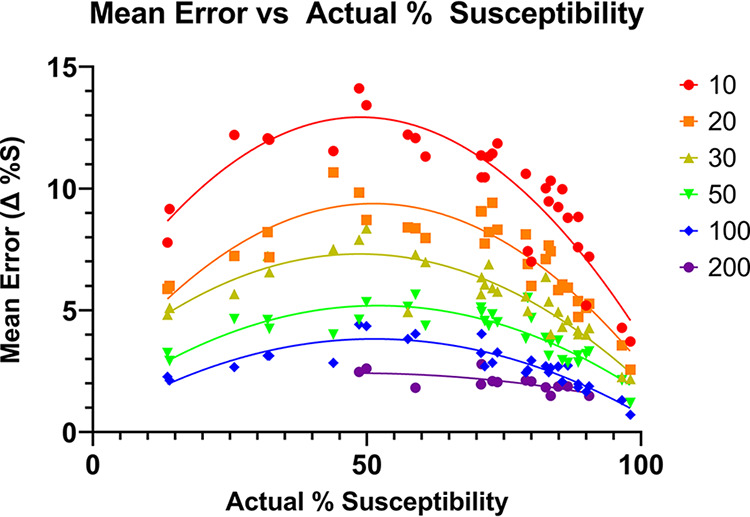
Observed mean errors for randomized subsets at the indicated sample size (color coded) as a function of actual % susceptibility, randomization from real data for calendar year 2020.

The mean and maximum errors were highly correlated with both the true percent susceptibility (higher mean and maximum error closer to 50%, lower near 0 or 100%), and the number of isolates (higher mean and maximum error with lower numbers of isolates). The mean errors (Fig. 1) ranged from 0.71% to 14.1%. For the random sample of 10, the mean error ranged from 3.72% to 14.11% (SD = 2.53, 95% CI = 8.91 to 10.73). For the random sample of 20, the mean error ranged from 2.56% to 10.66% (SD = 1.82, 95% CI = 6.46 to 7.78). For the random sample of 30, the mean error ranged from 2.18% to 8.36% (SD = 1.47, 95% CI = 4.97 to 6.03). For the random sample of 50, the mean error ranged from 1.20% to 5.64% (SD = 1.03, 95% CI = 3.60 to 4.35). For the random sample of 100, the mean error ranged from 0.71% to 4.42% (SD = 0.86, 95% CI = 2.39 to 3.04). For the random sample of 200, the mean error ranged from 1.49% to 2.79% (SD = 0.38, 95% CI = 1.82 to 2.26).

**FIG 2:**
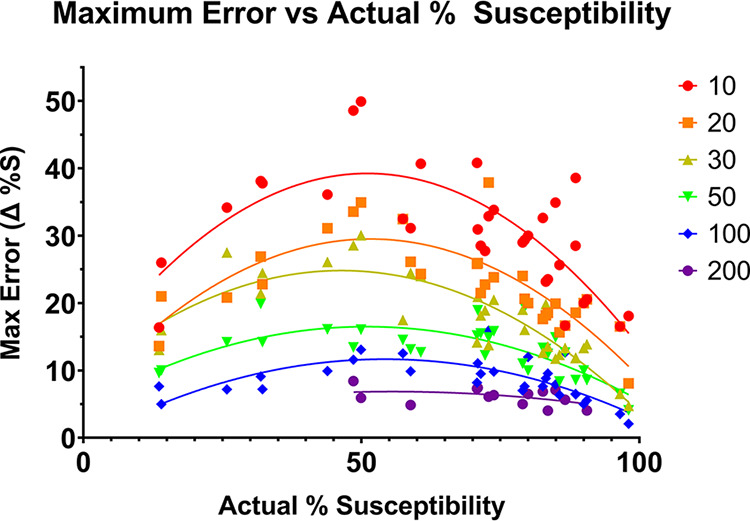
Maximum error observed among 50 replicates for randomized subsets at the indicated sample size (color coded) as a function of actual % susceptibility, randomization from real data for calendar year 2020.

The maximum errors (Fig. 2) ranged from 2.08% to 49.9%. For the random sample of 10, the maximum error ranged from 16.4% to 49.9% (SD = 8.61, 95% CI = 27.3 to 33.5). For the random sample of 20, the maximum error ranged from 8.08% to 37.9% (SD = 6.42, 95% CI = 20.2 to 24.8). For the random sample of 30, the maximum error ranged from 4.74% to 30.1% (SD = 6.20, 95% CI = 15.3 to 19.7). For the random sample of 50, the maximum error ranged from 4.08% to 19.9% (SD = 3.67, 95% CI = 11.4 to 14.1). For the random sample of 100, the maximum error ranged from 2.08% to 15.9% (SD = 3.03, 95% CI = 7.41 to 9.67). For the random sample of 200, the maximum error ranged from 4.04% to 8.42% (SD = 1.29, 95% CI = 5.37 to 6.86).

**FIG 3:**
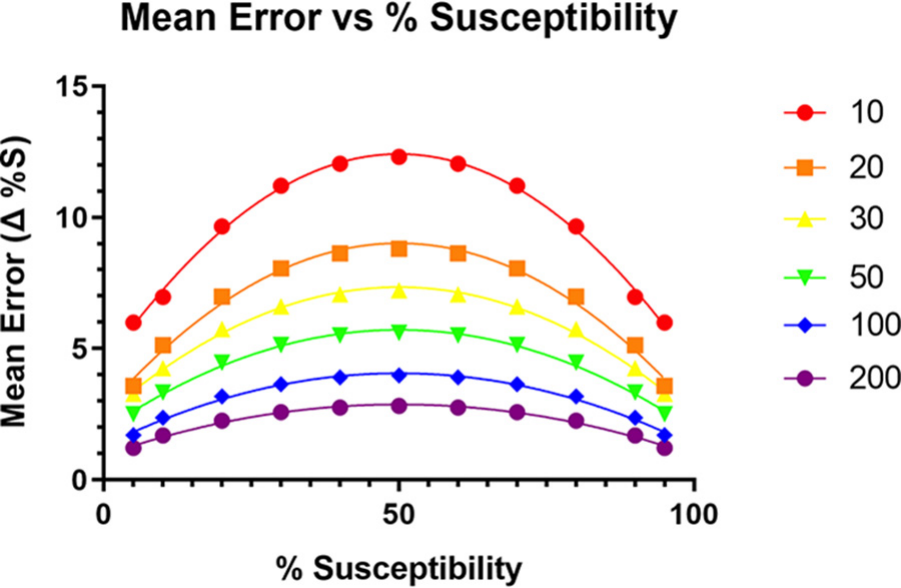
Mean error predicted using a binomial distribution function at the indicated sample size (color coded) as a function of % susceptibility.

Data generated using the binominal distribution function correlated well with the average errors estimated in the simulation (binomial distribution in Fig. 3; compare with Fig. 1). Binomial distributed data were also used to determine the sample sizes required to average ≤5% error at various % true susceptibility. A 30 isolate sample size was adequate to achieve this average level of error when true %S was between 0% and 10% or 90% to 100%, but >60 isolates are required when true %S is between 40% and 60% (Table 1).

**TABLE 1:** Number of isolates needed to achieve ≤5% average error at % susceptibility

	% susceptibility												
	5%	10%	15%	20%	30%	40%	50%	60%	70%	80%	85%	90%	95%
Isolates	14	24	33	40	54	62	64	62	54	40	33	24	14

### Comparison of 2-year aggregate data versus single year data.

**FIG 4:**
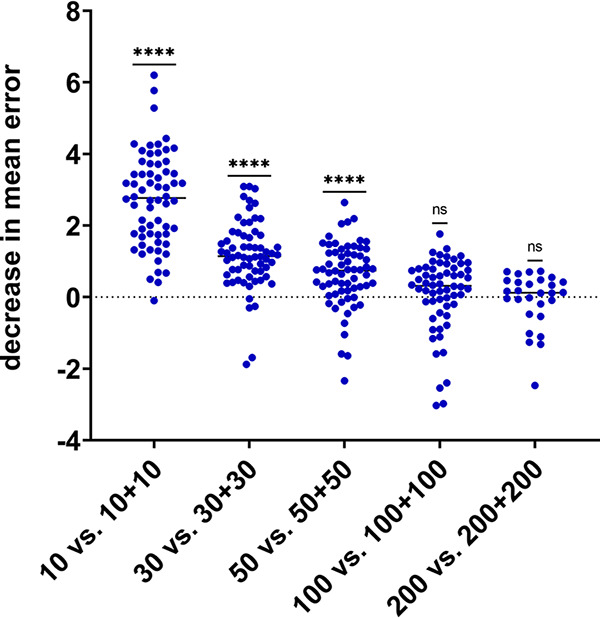
Decrease in mean error observed when doubling sample size by combining n+n results from adjacent years compared to n results from a single year.

Twenty-eight to 64 measurements were made for 2-year aggregate compared to a single year difference in mean error for each eligible data set from 2018 to 2020 data (Fig. 4). Two-year aggregate data had significantly lower mean error for sample sizes of 10, 30, and 50 (*P* < 0.0001), but not 100 (*P* = 0.07) or 200 (*P* = 0.68).

Ciprofloxacin results were included in the analysis above but it worth noting that a change in the CLSI breakpoints for fluoroquinolones was implemented during 2020 (June) which led to an artificial decrease in susceptibility for that year that was greater than would be expected for year-over-year change due to natural increase in resistance. For example, from 2019 to 2020 the true susceptibility rate of Klebsiella pneumoniae for ciprofloxacin decreased from 88.2% to 83.5%; in the same time period the susceptibility rate for Escherichia coli decreased from 76.8% to 70.1%. CLSI recommends with a breakpoint change mid-year to re-analyze the entire year of data using the new breakpoints but this was not possible because the commercial susceptibility testing system previously did not include low enough drug concentrations to use the modified breakpoints and were changed when an updated AST panel was implemented. Results were included as reported (S/I/R) for this reason. Of note the single data point at *n* = 10 in Fig. 4 for which aggregate 2-year data did not have a decrease in mean error compared to a single (latter) year data were for sampling Klebsiella pneumoniae and ciprofloxacin across 2019 to 2020 data.

### Year-over-year change as a function of isolate count.

**FIG 5:**
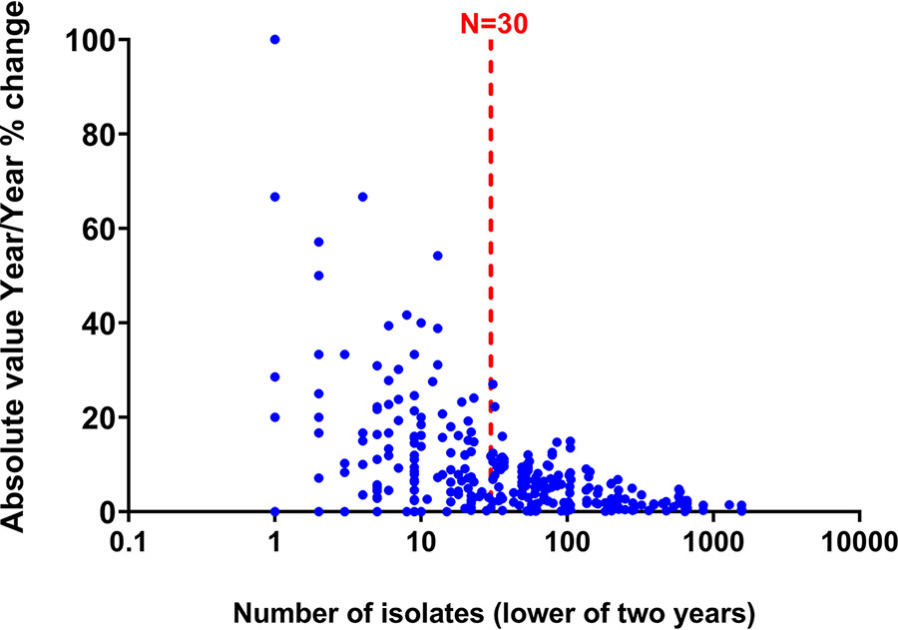
Year-over-year change as a function of isolate count for organism-drug combinations with expected susceptibility results between 10% and 90% (2020 versus 2021).

For organism-drug combinations with expected susceptibility results between 10% and 90% and lower numbers of isolates large variations from 1 year to the next were observed, as would be expected, although the scale of variation even for organism-drug combinations with at least 30 isolates was greater than expected (Fig. 5). The *x* axis value is the lower isolate number from the 2 years considered, all organism-drug combinations had similar numbers of isolates for the 2 years, but at very low counts some were discordant (for example 0/1 S 2020 versus 2/7 S 2021, 28.6% difference although listed as one isolate).

## DISCUSSION

Cumulative antibiograms provide important information to health care providers regarding the management of infectious diseases. The CDC Core Elements of Hospital Antibiotic Stewardship Programs (https://www.cdc.gov/antibiotic-use/healthcare/pdfs/hospital-core-elements-H.pdf [6]) note that facility-specific treatment guidelines may be based on national guidelines but should reflect hospital treatment preferences based on local susceptibilities, formulary options, and patient mix. At our institution, the antimicrobial stewardship program maintains an Empiric Therapy for Positive Blood Cultures Guideline, for example. Our policy is to recommend empirical agents as monotherapy only if our institutional antibiogram suggests greater than 90% susceptibility. If the antibiogram reports <90% susceptibility then an alternative empirical agent is recommended or a second antimicrobial is added for double coverage. As such, small changes in susceptibility reporting year to year may significantly impact our guideline recommendations. The Infectious Diseases Society of America (IDSA) also produces guidelines which make reference to using local antibiogram data. For example, the IDSA document “Guidance on the Treatment of Antimicrobial-Resistant Gram-Negative Infections” recommends that physicians consider local susceptibility patterns for the most likely pathogens when determining empirical therapy for a given patient (https://www.idsociety.org/practice-guideline/amr-guidance-2.0/), and the IDSA guideline for management of adults with hospital-acquired and ventilator-associated pneumonia utilize the local antibiogram data for S. aureus (methicillin resistance) a decision branch in the selection of empirical therapy (7). Errors in antibiograms can result in clinicians selecting inappropriate empirical antibiotics and in antimicrobial stewardship programs making inappropriate recommendations in institutional guidelines. Ineffective antibiotics can result in poor patient outcomes, while unnecessary broad-spectrum agents can promote antimicrobial resistance (8). Additionally, year-to-year variation in % susceptibility could result in antimicrobial stewardship and infection control programs inappropriately interpreting change as an outcome of their interventions. Prior literature, as well as analysis of publicly available antibiograms, demonstrates that antibiograms often include data points with fewer isolates than what is recommended (minimum 30), which is why we included evaluation of as few as 10 to 20 isolates for random sample sizes. This may occur because health care facilities, or those serving subsections or specific populations (cancer, ICU, pediatric, etc.) of health care facilities, request location or patient-group specific antibiogram services despite having too few patient isolates to populate an acceptably accurate antibiogram. For example, the CDC “Core Elements of Antibiotic Stewardship for Nursing Homes” (http://www.cdc.gov/longtermcare/index.html [9]) document and checklist recommends that long-term-care facilities request location specific antibiograms, although most such facilities do not generate enough isolates for creation of antibiograms that meet CLSI recommendations.

We sought to demonstrate the potential impact of using low numbers of isolates on the average (mean) and maximum errors in % susceptibility in antibiograms using a simple mathematical simulation of actual AST data and to determine how this correlates with binominal function distributions. Mean error was relatively high, particularly near true susceptibility rates of 50%, for data points using less than 30 isolates. Even when following the current recommendation, where a data point consisting of 30 isolates is acceptable, this study predicts a mean error of +/– 7% for data points near 50% susceptibility, which is in line with what was observed in year-over-year variability for combinations in this range (Fig. 1; Fig. 3). Given the relatively large expected error rates predicted in this study, and results that demonstrate 2-year aggregate data may be more accurate than single-year data for isolate numbers of 30 or less, a potential solution is to routinely include more than 1 year of data for organisms with fewer than 50 or 100 isolates, unless there is some reason to expect significant year-over-year changes in susceptibility. The study has some important limitations. One limitation of this study is that by its nature, only organism/drug combinations for which there are large sample sizes were analyzed. The results may not present a good model for results from organisms which routinely have fewer isolates, even at large institutions, especially if there is some unknown factor unique to particular organisms with low isolate numbers that affects susceptibility differently than a random sample from common organisms with a large number of isolates would. This limitation means that, in effect, this study only assesses the most common bacteria/drug combination but does not assess other organisms, such as fungi. Of note, a greater maximum error near 50% susceptibility, where divergence of a random sample from the true susceptibility is more likely to occur, was observed; in theory the largest possible errors would occur near the poles (near 0% or 100% susceptible) but would be observed less frequently. This study provides an important discussion point for further work evaluating the impact of data set size on the generalizability of antibiogram data.

### Conclusion.

This study assesses the potential level of error in antibiograms by using simulated results of different sample sizes from a larger pool of susceptibility tests for 32 different organism/drug combinations across 3 years of local antibiogram data, with comparison to theoretical data based on binomial distributions. Additionally, it provides insight into the potential of using aggregate data from 2 years as an alternative for organism/drug combinations where the sample size from a given year is particularly small. The findings of this study are supportive of the current recommendation of *at least* a 30-isolate cutoff for inclusion in cumulative antibiograms and provide some context about the expected level of year-over-year change in % susceptibility that may be attributable to limited sampling rather than actual changes in resistance rates.

## MATERIALS AND METHODS

### Susceptibility testing methods.

Antimicrobial susceptibility testing results were generated with a variety of methods, primarily an FDA cleared automated broth based system (Phoenix, Becton, Dickinson, PMIC-106 Gram-positive panel, NMIC-300 and NMIC-311 Gram-negative panels) or Kirby-Bauer disk diffusion, with a very small number of results using gradient diffusion strips (Etest, bioMérieux). Over 95% of results were generated using an automated broth based system, with other methods used only to verify results or assess unusual organism/drug combinations. When results were checked with a separate method for confirmation or because a resistance profile was atypical, only the final accepted result was used for analysis. All sample types were included in this study, as is common practice for antibiogram generation.

### Random sampling.

Susceptibility results from a total of 32 organism/drug combinations at two different collecting institutions (testing performed in a single hospital laboratory), were obtained for 2018, 2019, and 2020 via Qlik and EPIC EMR/EPIC Beaker LIS (Table 2). The CLSI recommendations for these time periods were followed: only diagnostic isolates with final verified results for routinely reported antibiotics were included, and only the first isolate of a given organism per patient per time period were included (repeat isolates excluded). The data were separated by year and drug. Organism/drug combination evaluated in this study were selected on the basis that the total number of isolates per combination was at least twice as much as the largest random sample size. In effect, this means that for a random sample of 200 isolates, the total pool would have to consist of at least 400 isolates. Using Microsoft Office Excel software, the RANDARRAY function was then used to assign a randomly generated number to each data point, and the INDEX function was used to rank the samples in order of their assigned numbers, resulting in a random sampling of the larger data set.

**TABLE 2:** Organisms analyzed, range of number of isolates for which results were reported (varied year to year and drug to drug), and the drugs for which results were analyzed

Organism	Institutions (range of isolates)	Drugs
E. cloacae	TJUH (220 to 238)	Ciprofloxacin, piperacillin-tazobactam, cefepime
E. coli	TJUH (1575 to 1748), MHD (631 to 763)	Ciprofloxacin, ceftriaxone, ampicillin
E. faecium	TJUH (204 to 213)	Ampicillin, linezolid, vancomycin
K. pneumoniae	TJUH (622 to 694), MHD (209 to 229)	Ciprofloxacin, piperacillin-tazobactam, ceftriaxone
MRSA	TJUH (301 to 318)	Clindamycin, erythromycin
MSSA	TJUH (462 to 530), MHD (173 to 196)	Clindamycin, erythromycin
P. aeruginosa	TJUH (516 to 896)	Cefepime, ciprofloxacin, piperacillin-tazobactam
P. mirabilis	TJUH (286 to 311)	Ceftriaxone, ciprofloxacin
S. epidermidis	TJUH (246 to 339)	Oxacillin, clindamycin

### Data processing.

Using the random sample generator, data sets of sizes 10, 20, 30, 50, 100, and 200 were obtained. Data sets were only generated when the total sample size was at least twice as large as the random sample size (e.g., total samples had *n* ≥ 400 in cases where random samples of 200 were obtained, data set sizes shown in Table 2). This process was repeated for 50 iterations, so that there were 50 simulated susceptibility rates for each data set size category. The actual susceptibility rate for the total data set was used for comparison. The error between the actual rate and the simulated rates were calculated for each iteration, and from that the mean error over all 50 iterations was found for each sample size category. Additionally, the maximum errors (largest error out of the 50 iterations) from the 2020 data for each sample size category were recorded.

### 2-year aggregate data.

Aggregate data from two consecutive years were obtained by combining susceptibility data from a random sample from each year, resulting in a combined aggregate sample equal to double the data set sizes detailed above (e.g., 10 randomly selected results from 2018 and 10 randomly selected results from 2019 combined, compared to 10 randomly selected from a single year, 50 iterations, evaluated for *n* = 10, 30, 50, 100, 200). The errors and mean error per sample size were calculated using the actual susceptibility rate from the latest year used in the aggregate data. Additionally, the difference between the latter consecutive year mean error and 2-year aggregate mean error was calculated (i.e., [2020 mean error] – [2019 to 2020 aggregate mean error]).

### Year-over-year change as a function of isolate count.

Data were extracted to Qlik via EPIC Beaker LIS from two consecutive calendar years (2020 and 2021) for three separate hospitals whose microbiology cultures and AST are performed by the institution’s central laboratory. Organism drug combinations which would be considered for inclusion in a cumulative antibiogram if sufficient numbers were available (many uncommon organisms were not included because they were never built in Qlik, even for the largest reporting unit hospital), but excluded organism drug combinations that were expected to be <10% or >90% susceptible based on prior antibiogram results. Data were included for each combination that had at least one isolate with AST results in each of the 2 years.

### Average error prediction using binominal distribution.

In addition to simulating the data sets by random sampling, expected values using a binominal distribution were calculated. The BINOM.DIST (binominal distribution) function in Microsoft Office Excel, along with the isolate count and susceptibility rate, were used to find the probability of distinct magnitudes of error occurring, and the contribution of those specific errors to average error. The binomial distribution can be used to predict the rate at which a particular result (susceptible [S] versus resistant [R] in this study) will occur given the number of independent experiments (isolate count) and probability (%S). As a potentially useful contextual point of reference for evaluating year-over-year change in %S in antibiograms, this mathematical approach was also used to determine the isolate count needed to achieve ≤5% average error for a given susceptibility rate.

### Statistical analysis.

The data were analyzed using GraphPad Prism. Mean and maximum error rates were plotted against the true susceptibility rates, and quadratic lines of best fit were created for each sample size category. For comparison of 2-year aggregates and 1-year random samples, Shapiro-Wilk tests were run to check the normality of the data sets, which were mostly not normally distributed; thus, Wilcoxon signed rank tests were used to analyze the difference in mean errors.
